# Left Ventricle Structure and Function in Young Adults Born Very Preterm and Association with Neonatal Characteristics

**DOI:** 10.3390/jcm10081760

**Published:** 2021-04-18

**Authors:** Adrien Flahault, Gabriel Altit, Aurélie Sonea, Anne-Sophie Gervais, Muhammad Oneeb Rehman Mian, Rong Wu, Eva Desbrousses, Ly Mai, Anik Cloutier, Jessica Simoneau, Anie Lapointe, Andréanne Villeneuve, Patrick Garceau, Michel White, Jean-Luc Bigras, Thuy Mai Luu, Anne Monique Nuyt

**Affiliations:** 1 Sainte-Justine University Hospital Research Center Sainte, Justine University Hospital, Faculty of Medicine, University of Montreal, Montreal, QC H3T 1C5, Canada; adrien.flahault@umontreal.ca (A.F.); aureliesonea@gmail.com (A.S.); anne-sophie.gervais@umontreal.ca (A.-S.G.); oneeb.mian@gmail.com (M.O.R.M.); rongwu60@hotmail.com (R.W.); eva.desbrousses@gmail.com (E.D.); lymai118@gmail.com (L.M.); anik.cloutier@recherche-ste-justine.qc.ca (A.C.); jean-luc_bigras@ssss.gouv.qc.ca (J.-L.B.); thuy.mai.luu@umontreal.ca (T.M.L.); 2 Division of Neonatology, Department of Pediatrics, Montreal Children’s Hospital, McGill University, Montreal, QC H3T 1C5, Canada; gabriel.altit@mail.mcgill.ca (G.A.); jessica.simoneau@muhc.mcgill.ca (J.S.); 3 Department of Pediatrics, Sainte-Justine University Hospital, Faculty of Medicine, University of Montreal, Montreal, QC H3T 1C5, Canada; lapointe_anie@hotmail.com (A.L.); andreanne.villeneuve@umontreal.ca (A.V.); 4 Department of Medicine, Montreal Heart Institute, Faculty of Medicine, University of Montreal, Montreal, QC H1T 1C8, Canada; patrick.garceau@gmail.com (P.G.); m_white@icm-mhi.com (M.W.)

**Keywords:** preterm birth, left ventricle, echocardiography

## Abstract

Preterm birth increases risk of cardiovascular disease and early death. A body of evidence suggests left ventricle (LV) echocardiographic alterations in children and adults born preterm. We aimed to determine if neonatal characteristics were associated with alterations in LV structure and function in preterm adults. We evaluated a cohort of 86 young adults born preterm below 30 weeks of gestation, and 85 full-term controls. We determined LV dimensions and function using tissue Doppler imaging, conventional and speckle tracking echocardiography (STE). Adults born preterm had smaller LV dimensions, but these differences did not remain after adjustment for body surface area (BSA), which was smaller in the preterm group. Stroke volume and cardiac output were reduced even after adjustment for BSA. We found a smaller e’ wave in the preterm group, but other markers of systolic and diastolic function did not differ. Use of antenatal steroids may be associated with a further reduced cardiac output in those born preterm. Adults born preterm show alterations in markers of LV dimensions and function. Identification of these markers may represent opportunities for early prevention of cardiovascular events in this at-risk population.

## 1. Introduction

Every year, 1% of all neonates are born below 30 weeks of gestational age (GA) [1]. Major advances in neonatal care in the past decades have significantly improved their survival, with the majority of them now reaching adulthood [2]. However, preterm birth impacts organ development, including the cardiac and vascular structures. During fetal life and until 36 weeks of gestation, the heart grows by active proliferation of cardiomyocytes. The rate of cardiomyocytes’ proliferation declines progressively from 20 to 38 weeks of gestation, following which the heart cells mature by hypertrophy [3]. The third trimester is therefore essential for optimal cardiac development. Preterm birth leads to a marked reduction in cardiomyocyte proliferation, which may adversely impact the cardiovascular structures and function over the life course [4]. Indeed, individuals born preterm are at a higher risk of early-onset heart failure [5], coronary heart disease [6], and early death from cardiovascular causes [7].

Studies analyzing echocardiography images have shown abnormalities in the cardiac structure and function of infants and children born preterm, from early post-natal life [8] throughout infancy [9,10,11] and in childhood [10,12,13]. In young adults born preterm, a number of studies have evaluated cardiac structure and function [14,15,16,17,18], and a recent meta-analysis [19] has shown increased left ventricle ejection fraction (LVEF), decreased LV stroke volume, and LV end-diastolic volume (indexed to body surface area, BSA), smaller e’ wave, a marker of diastolic dysfunction. In this study [19], LV mass was lower in children, but not in adults. Studies conducted in fetuses, infants, and children show that the use of antenatal steroids [20,21,22], bronchopulmonary dysplasia [23], preeclampsia [24] and intrauterine growth restriction [25] may alter cardiac development, while a study has shown a reduction in longitudinal peak systolic strain in adults born preterm after a diagnosis of preeclampsia during pregnancy [14]. Overall, scarce data is available on association of cardiac alterations in adulthood with events from the neonatal period. In this report, we aimed to assess the association of prematurity and neonatal complications on adult LV structure and function. As part of the Health of Adult born Preterm Investigation (HAPI) study, we compared echocardiographic characteristics of adults born preterm ˂ 30 weeks of GA to adults born full-term. We then assessed the association of LV parameters previously shown to be altered in the preterm population with neonatal characteristics and comorbidities.

## 2. Materials and Methods

### 2.1. Study Population

Data were obtained from the HAPI study, which has been previously described [26,27]. We obtained data from a cross-sectional observational study, the HAPI project, that evaluated the health of young adults (18–29 years) born at ≤29 weeks GA between 1987 and 1997 and compared them to individuals born full-term (≥37 weeks GA) matched for sex and age (±2 years) and recruited among friends and siblings. Participants from the preterm group were admitted to one of the three main neonatal intensive care units in Montreal, Quebec: Sainte-Justine University Hospital (Centre Hospitalier Universitaire Sainte-Justine (CHUSJ)), and the McGill University affiliated Royal Victoria Hospital and Sir Mortimer B. Davis Jewish General Hospital. Ethics approval was obtained from Sainte-Justine University Hospital, McGill University Health Centre, and Sir Mortimer B. Davis Jewish General Hospital Research Ethics Boards. Participants from the pilot phase of the study were excluded since they did not all receive a complete echocardiographic assessment. All participants gave written informed consent to participate in the study.

### 2.2. Current Clinical Characteristics

Height and weight were measured in duplicates, on study day. BSA was calculated using the Mosteller formula (square root of the height (cm) multiplied by the weight (kg) divided by 3600) [28]. Office blood pressure and heart rate measurements on the study day were performed as previously described [26]. Level of education and current smoking were obtained using a questionnaire.

### 2.3. Neonatal Characteristics and Comorbidities

Neonatal information was collected from medical files (preterm group) and immunization booklets (term group). Birth weight for gestational age percentile was calculated according to Hadlock et al. [29] (preterm group) or to Kramer et al. [30] (term group). Small for gestational age was defined as birth weight below the 10th percentile. Moderate to severe bronchopulmonary dysplasia (BPD) was defined as supplemental oxygen use after 36 weeks post-menstrual age in those born preterm, per 2001 NICHD consensus workshop definitions [31].

### 2.4. Cardiac Imaging

Echocardiographic views were obtained at rest in the left lateral decubitus position on a General Electric (GE) Vivid E9 ultrasound machine with standard transducers, and standard echocardiographic measurements were extracted and analyzed using EchoPac PC software (both from GE Healthcare; Chicago, IL, USA). Offline measurements were done on Tomtec Arena platform (Version 4, Unterschleissheim, Germany, 2017). Measurements were performed according to the American Society of Echocardiography guidelines [32,33]. Cardiac cycle was defined by peak R to R from electrocardiogram integration. End-systole was defined by smallest ventricular volume. Mitral valve inflow velocities were obtained from pulsed-wave Doppler imaging. Tissue Doppler imaging at the lateral LV wall was obtained as a single measure in the apical-4-chamber view. Speckle-tracking echocardiography (STE), allowing for estimation of global systolic and diastolic deformation by following the endocardial border [34], was used to evaluate the strain (magnitude of deformation in %) and strain rate (speed of deformation in 1/s). Images of the apical views, as well as the parasternal short-axis view at the level of the papillary muscle of the mitral valve were uploaded on the Tomtec Arena platform for STE assessment. Endocardial tracing was done manually, point-by-point, at end of systole and diastole. Peak global longitudinal systolic strain and strain rate, as well as global circumferential and radial strain and strain rate were collected from the output. Peak diastolic e’ strain rate (early diastolic peak) value was extracted from the average strain rate curve in the apical-4-chamber view analysis [35,36]. Tomtec Arena also provides an automated estimation of end diastolic (EDV) and end systolic volume (ESV) from the tracing in the apical views, from which it estimates ejection fraction. All measurements were extracted by experienced sonographers blinded to the group assignment. For Peak Global Longitudinal Strain, all 3 views (apical 2 chambers, 3 chambers, and 4 chambers) were used for analysis when available. Otherwise, the maximal views were included in the analysis. Mean ± standard deviation frame rates for STE were 64 ± 9 frames per second. Velocity time integral (VTI) of the LV outflow tract estimates red blood cell stroke distance traveled per cardiac contraction, as a surrogate measure for output in the corresponding vessel [37]. Left ventricular outflow tract (LVOT) VTI of the pulsed wave Doppler, measured at the level of the aortic valve attachments, was measured in the apical 3 chamber view. Aortic valve (AV) was measured in the parasternal long-axis view. Calculated stroke volume was derived from the VTI multiplied by the corresponding outflow cross-sectional area: (AV/2)^2^ × Pi. The cardiac output (CO) was then estimated by multiplying the resultant stroke volume by the heart rate [38].

Inter-rater variability was assessed in a subset of 50 participants (25 born full-term and 25 born preterm) by two echocardiography experts (G.A., J.S.) blinded to the previously obtained values and to the exposure group for selected parameters. We determined consistency intraclass correlation coefficients (ICC) using a two-way mixed model. Inter-rater agreement was considered poor (ICC below 0.40), fair (ICC between 0.40 and 0.59), good (ICC between 0.60 and 0.74) or excellent (ICC above 0.75), according to Cicchetti [39].

### 2.5. Association with Neonatal Characteristics

Based on previous literature, we investigated the association of neonatal characteristics that were reported to be associated with LV changes (use of antenatal steroids [20,21,22], bronchopulmonary dysplasia [23], preeclampsia [14,24], and intrauterine growth restriction [24,40]) with LV parameters that were reported to be altered in preterms in a recently published meta-analysis [19] (LV mass, LVEF, LV E/A, LV e’ and cardiac output). Peak global longitudinal strain was not included in the meta-analysis but was previously shown to be associated with maternal preeclampsia in adults born preterm [14].

### 2.6. Statistical Analyses

Descriptive statistics were calculated as mean with standard deviation (SD) for continuous variables and counts with proportions for categorical variables. All between-group comparisons of cardiac volumes and dimensions were performed with and without adjustment for BSA, because of a significant difference in BSA between term and preterm groups and the well-known physiological association between BSA and cardiac dimensions. Univariate and bivariate linear regressions were performed to assess mean and adjusted mean differences, except for baseline characteristics (Table 1). Normality of residuals was assessed visually and using the Shapiro–Wilk test. We used the False Discovery Rate method to adjust *p*-values for multiple comparisons within all domains of interest. Our study has a >90% power to identify a difference of 0.5 SD between groups. *p* values < 0.05 were considered statistically significant. Number of missing data is provided in the tables. There was no missing data for BSA. All analyses were performed using R version 3.6.0 (International Open Source Collaborative, R Core Team, Vienna, Austria) [41].

## 3. Results

### 3.1. Study Population

In this study, 85 participants born full-term (GA at birth ≥ 37 weeks) and 86 participants born preterm below 30 weeks GA were included. Among those born preterm, 28 (33%) had a history of moderate to severe BPD. Detailed neonatal and adult clinical characteristics of study participants are shown in Table 1.

### 3.2. Conventional Echocardiographic Markers of LV Dimensions and Systolic Function

Conventional markers of LV systolic function (ejection fraction by Simpson’s Biplane disc method and fractional shortening by M-Mode), were preserved and similar in participants born at term and preterm (Table 2). Individuals born preterm had smaller LV dimensions by 2D measurements and M-mode, both in diastole (Table 2) and systole (Supplementary Table S1). Automated estimation of EDV parameters by speckle-tracking echocardiography showed significantly decreased volumes in the preterm group before adjustment for BSA. However, these differences were no longer present after adjusting for BSA (Table 2). Similarly, LV mass was lower in the preterm groups, but the differences were no longer significant after adjustment for BSA (Table 2).

Heart rate was higher in the preterm group. Stroke volume and cardiac output were lower in the preterm group, even after adjustment for BSA (Table 3). Mitral valve Doppler and lateral tissue Doppler imaging did not show any difference in E/A ratio and E/e’ ratio. However, e’ wave that represents early diastolic filling, was significantly shorter in the preterm group, as well as LV S’ wave (Table 3). Echocardiographic measures of volume and mass are also shown indexed to BSA in Supplementary Table S1).

### 3.3. Speckle-Tracking Echocardiography Analysis

LV STE measurements could be performed in 87% of the study participants. Poor image quality did not allow for accurate measurements in the remaining participants. We found no significant difference among the analyzed strain and strain rate parameters in systole or diastole (Table 4 and Supplementary Table S2).

### 3.4. Associations with Antenatal and Neonatal Characteristics

In the preterm group, we did not find a significant association of maternal preeclampsia, birth weight percentile and bronchopulmonary dysplasia on LV mass, LVEF, LVCO, LV E/A, LV e’, and peak longitudinal strain. Use of antenatal steroids was associated with a lower LVCO in the preterm group (Table 5).

### 3.5. Sex-Stratified Comparisons

We performed analyses of LV echocardiography markers stratified by sex and found that the differences observed between term and preterm adults were similar among males and females (Supplementary Table S3).

### 3.6. Reproducibility

All measurements had good or excellent reproducibility. Specifically, ICC was of 0.81 for S’, 0.86 for e’, 0.90 for EDV and 0.89 for LV mass.

## 4. Discussion

Using echocardiography-derived markers of dimension and function, we report characterization of the LV in a large cohort of young adults born preterm < 30 weeks, compared to term counterparts. Overall, LV estimates of volumes as measured by echocardiography were decreased in the preterm population but did not remain after adjustment for BSA. LV mass, when adjusted for BSA, was similar between groups. While most markers of cardiac function were preserved in those born preterm, we observed a lower stroke volume, a smaller LV e’ wave and a smaller s’ wave in the preterm group, in line with previous reports. LV length was also shorter in the preterm group. Use of antenatal steroids may be associated with a further decreased cardiac output.

As shown by previous studies and a recent meta-analysis, children and adults born extremely preterm who survive the neonatal period display mild but significant echocardiographic alterations of systolic and diastolic function [19]. Most of the results from our cohort are in line with this meta-analysis, which included 6 studies of a total of 301 young adults (mean ages 18–25 years) born preterm, compared to 281 full-term controls. Indeed, we found a lower e’ wave, a similar LV mass (when accounting for BSA), a similar S’ wave, a similar LV strain and a similar LV E/A ratio. While the meta-analysis reports a slightly higher LVEF in those born preterm < 32 weeks, as well as a decreased LVEDV when indexed to BSA, we found no difference in these parameters when accounting for BSA. A trend towards a lower LVEDV was observed in our study, however, suggesting the lack of difference may be explained by a lack of power. In addition, we found, in line with the meta-analysis, a lower stroke volume in the preterm group, even after adjustment for BSA. Cardiac output was also lower in the preterm group. In contrast, heart rate was higher in the preterm group. Cardiac output did not differ between groups when after adjustment for BSA, while stroke volume remained lower after adjustment for BSA. Hence, it is likely that the increase in heart rate among preterms allows maintaining a preserved cardiac output, compensating for the smaller LV. Although isolated, LV e’ alteration may be of clinical importance. Indeed, early diastolic peak velocity (e’) predicts mortality in patients with cardiac diseases [42,43]. Preterm birth has been associated with systemic hypertension in adulthood [27,44], as well as with an increased risk of cardiovascular events [6] and related mortality [7]. Of note, although e’ was lower in the preterm group, we found no difference in early diastolic strain rate. This may be explained by the fact that there was a higher number of missing data for deformation analysis, leading to a lack of power for this parameter. Indeed, a previous study has found alterations in strain parameters in adults born preterm from a pregnancy complicated by preeclampsia [14]. TDI has a high temporal resolution and only reflects longitudinal assessment on the myocardium just below the base. The strain rate takes into account all the segments of the ventricle. Thus, our results could also suggest that the LV alterations observed in the preterm group mainly concern the sub-mitral area or LV free wall area.

Two recent studies were not included in the meta-analysis. Similar to our finding, Harris et al. [18] found a lower LV stroke volume and cardiac output, but no difference in LVEF, in low birth weight adults, including 25% born before 28 weeks GA. Goss et al., using magnetic resonance cardiac imaging in 38 adults born preterm (mean GA, 29 weeks), also found a reduced LV stroke volume, but was associated with hypercontractile strain which was not found in our study [17].

While our study, in line with other recent studies, suggests mild but long-term alterations of cardiac structure and function in adults born preterm, little is known on the association of these alterations with the adverse conditions associated with preterm birth. In an experimental study conducted in an experimental model that replicates some of the adverse conditions of preterm birth, we previously reported changes in LV structure and function, including increased susceptibility to heart failure in 12-week old rats that had been exposed to high (80%) oxygen levels from day 3 to 10 of life, compared to controls not exposed [45]. Furthermore, myocardial fibrosis was observed in animals exposed to high levels of oxygen in the neonatal period [45,46,47], which has been strongly associated with alterations in diastolic function in humans [48]. Bronchopulmonary dysplasia, a chronic lung disease characterized by prolonged supplemental oxygen requirement during preterm birth, has been associated with lower E/A ratio and lower e’ wave velocity in infants born preterm [23]. In contrast, we found no association of these parameters, or other parameters previously shown to be altered in adults born preterm, with bronchopulmonary dysplasia in our cohort of young adults born preterm.

Two studies from the 1990s performed around the period of birth of our study population, found alterations in LV filling and increased wall thickness in preterm neonates receiving postnatal administrations of steroids [21,22]. In contrast, a study conducted in children found no association between antenatal or postnatal steroid treatment and cardiac outcomes [20]. In our study, we suggest that antenatal steroid treatment may be associated with a lower cardiac output in young adults born preterm. This finding requires confirmation from other cohort studies or a meta-analysis.

Although a history of preeclampsia during pregnancy has been associated with alterations in LV strain in adult offspring born preterm [14], we did not find this association in our study.

Intrauterine growth restriction has been associated with different cardiac shape, subclinical systolic and diastolic changes in children, with decreased myocardial peak velocities, increased E/e’ ratio and E deceleration time, higher blood pressure, and increased intima-media thickness [25]. While we did find a higher diastolic blood pressure in those born preterm in this cohort, as previously reported [27], we found no association between echocardiographic parameters and percentile birth weight, as a marker for intra-uterine growth in our study. Similarly, we previously reported from the HAPI study a similar carotid intima-media thickness between those born preterm and full-term [49].

We found a smaller LV length in the preterm group. A shorter LV length was previously reported to be associated with an altered exercise systolic response in adults born preterm [15]. Thus, a shorter LV length may contribute to the altered exercise tolerance in this population.

Our study has limitations. First, although the number of very preterm individuals included in our study is relatively high and similar to previously reported cohorts, our study may not have the power to identify smaller associations of antenatal and perinatal factors with adult cardiac characteristics. Second, it is possible that changes observed between groups are the result of regional differences in neonatal care, including rates of antenatal steroids as suggested by current results, habitus, and/or genetic backgrounds. Our findings were obtained from mainly white individuals born in Quebec, which could limit generalizability. In addition, there is a strong inherited component to the risk of cardiac diseases, which the current study, as well as previous studies on the matter, did not have the power to take into account. In women, having delivered preterm (independently of hypertensive disorders of pregnancy) is a recognized risk factor for cardiovascular diseases [50]. Hence, a global collaborative effort to evaluate cardiovascular parameters homogeneously in different cohorts from different regions of the world may contribute to deepen our understanding of the determinants leading to long-term cardiovascular alterations following preterm birth.

Beyond these limitations, the current results add to the increasing evidence that individuals born preterm display altered LV structure and function, with a possible association with prematurity-associated conditions such as antenatal steroids.

## 5. Conclusions

Stroke volume, cardiac output, and e’ wave are decreased in young adults born preterm included in this study, and cardiac output may be further reduced in case of antenatal steroid use. The differences observed between groups were relatively small and overall measures of LV function were within normal ranges—fortunately so at such a young adult age. Whether the observed alterations translate into a higher risk of heart failure later in life and could be used to refine risk stratification remains to be determined.

## Figures and Tables

**Table 1:** Neonatal and Adult Characteristics.

	Term	Preterm	
	*n* = 85	*n* = 86	
	Mean ± SD or *n* (%)	Mean ± SD or *n* (%)	*p*-Value
Neonatal Characteristics			
Male sex, *n* (%)	36 (42)	38 (44)	0.88
White ethnicity, *n* (%)	76 (89)	79 (92)	0.61
Gestational age, weeks	39.6 ± 1.1	27.2 ± 1.4	-
Birth weight, g	3373 ± 363	963 ± 225	-
Birth weight percentile, %	46.5 ± 23.5	34.9 ± 16.9	<0.001
Small for gestational age, *n* (%)	7 (8)	6 (7)	0.78
Maternal preeclampsia, *n* (%)	6 (7)	19 (22)	0.008
Antenatal steroids, *n* (%)	0 (0)	35 (42)	-
Moderate to severe BPD, *n* (%)	-	28 (33)	-
Postnatal steroids, *n* (%)	0 (0)	26 (31)	-
Current Characteristics			
Age, years	23.2 ± 2.4	23.3 ± 2.3	0.86
Height, cm	170 ± 8	166 ± 9	<0.001
Weight, kg	69.4 ± 15.3	61.6 ± 11.8	<0.001
Body mass index, kg/m^2^	23.9 ± 4.6	22.4 ± 3.7	0.023
Body surface area, m^2^	1.80 ± 0.22	1.68 ± 0.19	<0.001
Education ≥ high school, *n* (%)	54 (69)	53 (67)	0.86
Current tobacco smoking, *n* (%)	16 (19)	20 (23)	0.57
SBP on study day, mmHg	116 ± 13	119 ± 14	0.097
DBP on study day, mmHg	68 ± 8	72 ± 9	0.002

BPD: bronchopulmonary dysplasia; SBP: systolic blood pressure; DBP: diastolic blood pressure. *p*-values are calculated using Student’s t-test or the Fisher exact test.

**Table 2:** Left Echocardiographic Structure and Function.

	Term		Preterm			
	*n* = 85		*n* = 86			
	Missing	Mean ± SD	Missing	Mean ± SD	*p*-Value	*p*-Value (Adjusted for BSA)
	*n* (%)		*n* (%)			
Simpson’s Disc Method Dimensions and Functional 2D Assessment						
LV Ejection Fraction (Biplane), %	12 (14)	56.8 ± 6.5	10 (12)	56.8 ± 6.8	0.94	-
LV length in diastole (A4C, mm)	12 (14)	83.9 ± 9	10 (12)	78.5 ± 8	0.001	0.27
EDV in A4C (mL)	12 (14)	112 ± 31.5	10 (12)	95.4 ± 29.7	0.004	0.59
EDV indexed in A4C, mL/m^2^	12 (14)	61.8 ± 11.8	10 (12)	56.3 ± 13.6	0.045	-
LV Dimensions and Function (M-Mode)						
Fractional shortening, %	1 (1)	33.7 ± 3.3	1 (1)	34.2 ± 3.6	0.38	-
LV internal diameter in diastole	1 (1)	47.5 ± 4.6	1 (1)	46 ± 3.9	0.048	0.85
LV posterior wall diameter in diastole	1 (1)	7.79 ± 1.28	1 (1)	7.57 ± 1.14	0.32	0.46
Interventricular septum in diastole, mm	1 (1)	7.06 ± 1.06	1 (1)	6.78 ± 0.8	0.091	0.85
LV mass, g	1 (1)	114 ± 30	1 (1)	104 ± 26	0.037	0.85
LV mass indexed, g/m^2^	1 (1)	63.1 ± 12.3	1 (1)	61.5 ± 11.6	0.63	-

A4C: apical 4-chamber view, EDV: end diastolic volume estimate, LV: left ventricle. *p*-values were computed after adjustment for multiple comparisons using the false discovery rate method. BSA: body surface area.

**Table 3:** Echocardiographic Markers of LV Function.

	Term		Preterm			
	*n* = 85		*n* = 86			
	Missing	Mean ± SD	Missing	Mean ± SD	*p*-Value	*p*-Value (Adjusted for BSA)
	*n* (%)		*n* (%)			
Heart rate, bpm	0 (0)	69.3 ± 12.7	0 (0)	75.4 ± 12.2	0.003	-
Left Ventricular Outflow Tract Doppler (Pulsed-Wave)						
Stroke volume, mL	4 (5)	66.8 ± 9.5	2 (2)	59.9 ± 9.3	<0.001	0.001
Stroke volume indexed, mL/m^2^	4 (5)	37.6 ± 5.9	2 (2)	36 ± 5.3	0.16	-
Cardiac output, L/min	4 (5)	4.43 ± 0.6	2 (2)	4.15 ± 0.51	0.003	0.27
Cardiac output indexed, L/min/m^2^	4 (5)	2.5 ± 0.4	2 (2)	2.51 ± 0.35	0.89	-
Doppler–Mitral Valve						
MV E/A ratio	1 (1)	1.8 ± 0.42	1 (1)	1.73 ± 0.38	0.26	-
E/e’ ratio	3 (4)	4.44 ± 0.8	1 (1)	4.59 ± 0.79	0.20	-
Tissue Doppler Imaging (Lateral)						
LV S’ wave (cm/s)	3 (4)	11.5 ± 2.3	1 (1)	10.7 ± 2.3	0.036	-
LV e’ wave (cm/s)	3 (4)	19.2 ± 2.6	1 (1)	17.7 ± 2.8	0.001	-

e’: early diastolic velocity of lateral wall of LV, E: peak early velocity of inflow pulsed-wave Doppler, A: atrial (late) peak velocity of inflow pulsed-wave Doppler, LV: left ventricle, MV: mitral valve, S’: peak systolic velocity of lateral wall of LV, VTI: velocity time integral. *p*-values were computed after adjustment for multiple comparisons using the false discovery rate method. BSA: body surface area.

**Table 4:** Speckle-Tracking Echocardiography.

	Term		Preterm		
	*n* = 85		*n* = 86		
	Missing	Mean ± SD	Missing	Mean ± SD	*p*-Value
	*n* (%)		*n* (%)		
Circumferential					
Circumferential Strain (%)	12 (14)	−29.8 ± 4.7	11 (13)	−29.9 ± 4.1	0.56
Circumferential Strain Rate (1/s)	12 (14)	−1.67 ± 0.3	11 (13)	−1.69 ± 0.29	0.80
Radial					
Radial Strain (%)	12 (14)	37.7 ± 14.9	11 (13)	36.1 ± 16.3	0.44
Radial Strain Rate (1/s)	12 (14)	1.52 ± 0.42	11 (13)	1.47 ± 0.48	0.47
Longitudinal					
Peak Longitudinal Strain LV-Apical 4 Chamber (%)	13 (15)	−21.8 ± 4.5	10 (12)	−21.7 ± 4.2	0.62
Peak Longitudinal Strain Rate LV-Apical 4 Chamber (1/s)	13 (15)	−1.13 ± 0.22	10 (12)	−1.11 ± 0.21	0.31
Peak Global Longitudinal Strain (%)	12 (14)	−21.1 ± 3.9	10 (12)	−21.3 ± 3.9	0.67

LV: left ventricle. *p*-values were computed after adjustment for multiple comparisons using the false discovery rate method. BSA: body surface area.

**Table 5:** Effect of neonatal factors on preterm adult left heart structure and function.

	Antenatal Steroids	Preeclampsia	Birth Weight Percentile, per 10%	Moderate to Severe Bronchopulmonary Dysplasia
	β (95% CI)	β (95% CI)	β (95% CI)	β (95% CI)
LV mass, g	−12.2 (−23.5, −0.99)	1.92 (−11.5, 15.3)	2.84 (−0.4, 6.09)	−10.3 (−21.9, 1.42)
LV EF, %	0.84 (−2.26, 3.94)	0.84 (−2.86, 4.53)	0.18 (−0.72, 1.08)	−0.53 (−3.81, 2.75)
LV CO, L/min	−0.31 * (−0.52, −0.09)	−0.14 (−0.40, 0.12)	0.02 (−0.05, 0.08)	−0.10 (−0.33, 0.13)
LV E/A	0.02 (−0.15, 0.18)	0.01 (−0.19, 0.20)	0.03 (−0.02, 0.08)	0.01 (−0.16, 0.18)
LV e’, cm/s	−0.29 (−1.54, 0.95)	−0.21 (−1.65, 1.23)	−0.02 (−0.38, 0.33)	−0.56 (−1.81, 0.7)
Peak Global Longitudinal Strain (%)	−0.18 (−1.96, 1.60)	−0.83 (−2.92, 1.26)	−0.08 (−0.59, 0.43)	0.22 (−1.64, 2.08)

LV: left ventricle; EDV: end diastolic volume. Results shown are adjusted mean differences obtained from coefficients B with 95% confidence interval (CI) calculated using linear regression. Only participants born preterm were included in this analysis. * *p* < 0.05 after adjustment for multiple comparisons.

## Data Availability

Data available upon request to the corresponding author.

## Supplementary Materials

The following are available online at https://www.mdpi.com/article/10.3390/jcm10081760/s1, Supplementary Table S1: Supplementary LV echocardiography markers. Table S2: Speckle-Tracking Echocardiography; Table S3: LV echocardiography markers according to sex and birth status.
