# Supplementary material for: Left Ventricle Structure and Function in Young Adults Born Very Preterm and Association with Neonatal Characteristics
Source: J Clin Med. 2021 Apr 18;10(8):1760. doi: 10.3390/jcm10081760 (PMC8072582; doi:10.3390/jcm10081760)
Supplement: Supplementary file 1 [file jcm-10-01760-s001.zip › jcm-1180124-supplementary.pdf]

Left ventricle structure and function in young adults born very preterm and association with neonatal characteristics

Table S1. Supplementary LV echocardiography markers.

	Term N=85		Preterm N=86			
	**Missing n (%)**	**Mean ± SD**	**Missing n (%)**	**Mean ± SD**	**p-value**	**p-value (adjusted for BSA)**
**Simpson's disc method dimensions and functional 2D assessment**						
LV length in systole (A4C), mm	12 (14)	66 ± 8.2	10 (12)	61.2 ± 8.5	0.007	0.345
ESV in A4C, mL	12 (14)	45.7 ± 16.2	10 (12)	39.7 ± 15.4	0.069	0.916
**Doppler - Mitral valve**						
MV deceleration time, ms	1 (1)	186 ± 34	1 (1)	182 ± 30	0.629	-
MV A wave duration, ms	1 (1)	117 ± 10	1 (1)	116 ± 9	0.72	-
LV IVRT, ms	3 (4)	56.5 ± 6.1	1 (1)	56.7 ± 6.1	0.888	-
LV IVCT, ms	3 (4)	60.1 ± 7.3	1 (1)	61.2 ± 9.3	0.629	-

A4C: apical 4-chamber view, ESV: end systolic volume estimate, LV: left ventricle. MV: mitral valve. IVRT: isovolumic relaxation time, IVCT: isovolumic contraction time. P-values were computed after adjustment for multiple comparisons using the False Discovery Rate method.

Table S2. Speckle-Tracking Echocardiography.

	Term N=85		Preterm N=86		
	**Missing n (%)**	**Mean ± SD**	**Missing n (%)**	**Mean ± SD**	**Mean difference**
**Circumferential**					
Peak Early Diastolic Circumferential Strain Rate, 1/s	12 (14)	2.11 ± 0.48	11 (13)	2.12 ± 0.5	0.02 (-0.14, 0.17)
**Longitudinal**					
Peak Longitudinal Strain LV – Apical 2 Chamber, %	15 (18)	-22.5 ± 4.7	16 (19)	-22.5 ± 5	0.03 (-1.58, 1.63)
Peak Longitudinal Strain LV – Apical 3 Chamber, %	23 (27)	-20.8 ± 4.6	20 (23)	-20.3 ± 4.7	0.48 (-1.14, 2.1)
Peak Longitudinal Strain Rate LV – Apical 2 Chamber, 1/s	15 (18)	-1.13 ± 0.27	16 (19)	-1.16 ± 0.29	-0.03 (-0.12, 0.06)
Peak Longitudinal Strain Rate LV – Apical 3 Chamber, 1/s	23 (27)	-1.07 ± 0.22	20 (23)	-1.03 ± 0.27	0.05 (-0.04, 0.13)
Peak Early Diastolic Longitudinal LV Strain Rate Apical 4 Chamber, 1/s	13 (15)	1.6 ± 0.39	10 (12)	1.58 ± 0.44	-0.02 (-0.16, 0.11)

LV: left ventricle. * P<0.05 after adjustment for multiple comparisons. Adjusted mean difference: mean difference after adjustment for body surface area.

Table S3. LV echocardiography markers according to sex and birth status.

	Term N=85		Preterm N=86		
	**Missing n (%)**	**Mean ± SD**	**Missing n (%)**	**Mean ± SD**	**p-value**
**Male**					
LV mass, g	1 (3)	129 ± 27	1 (3)	118 ± 25	0.090
LV EF, %	4 (11)	55.8 ± 6.5	5 (13)	56.9 ± 6.9	0.50
LV CO, L/min	3 (8)	4.47 ± 0.68	2 (5)	4.14 ± 0.55	0.030
LV E/A	1 (3)	1.8 ± 0.48	1 (3)	1.77 ± 0.39	0.75
LV e′, cm/s	2 (6)	19.3 ± 2.4	1 (3)	17.6 ± 3.1	0.012
Peak Global Longitudinal Strain (%)	4 (11)	-21.1 ± 3.9	5 (13)	-21.1 ± 4.5	0.98
**Female**					
LV mass, g	0 (0)	104.2 ± 28.1	0 (0)	92.5 ± 20.8	0.022
LV EF, %	8 (16)	57.6 ± 6.5	5 (10)	56.8 ± 6.9	0.59
LV CO, L/min	1 (2)	4.4 ± 0.55	0 (0)	4.16 ± 0.49	0.030
LV E/A	0 (0)	1.79 ± 0.38	0 (0)	1.69 ± 0.38	0.21

LV e', cm/s	1 (2)	19.1 ± 2.8	0 (0)	17.8 ± 2.6	0.019
Peak Global Longitudinal Strain (%)	8 (16)	-21.2 ± 3.9	5 (10)	-21.4 ± 3.3	0.87

LV: left ventricle; EDV: end diastolic volume. P values are calculated without adjustment for multiple comparisons.
